# Satisfaction and Beliefs on Gender-Based Violence: A Training Program of Mexican Nursing Students Based on Simulated Video Consultations during the COVID-19 Pandemic

**DOI:** 10.3390/ijerph182312284

**Published:** 2021-11-23

**Authors:** Diana Jiménez-Rodríguez, Oscar Arrogante, Maravillas Giménez-Fernández, Magdalena Gómez-Díaz, Nery Guerrero Mojica, Isabel Morales-Moreno

**Affiliations:** 1 Department of Nursing, Physiotherapy and Medicine, University of Almeria, 04120 Almeria, Spain; 2 Red Cross University College of Nursing, Spanish Red Cross, Autonomous University of Madrid, 28003 Madrid, Spain; oscar.arrogante@cruzroja.es; 3 Faculty of Nursing, Campus de los Jerónimos s/n, Catholic University of Murcia, Guadalupe, 30107 Murcia, Spain; mgimenez@ucam.edu (M.G.-F.); mgdiaz@ucam.edu (M.G.-D.); imorales@ucam.edu (I.M.-M.); 4 Department of Nursing, Autonomous University of Aguascalientes, Aguascalientes 20100, Mexico; g.n.ery@hotmail.com

**Keywords:** COVID-19, gender-based violence, high-fidelity simulation training, nursing education, qualitative research, video conferencing, virtual simulation

## Abstract

The increase in gender-based violence in light of the COVID-19 pandemic is a public health problem that needs to be addressed. Our study aimed to describe the satisfaction with a training program in gender violence victim’s attention through simulated nursing video consultations, analyze the beliefs on gender violence in Mexican undergraduate nursing students, and understand the skills that need to be improved. A descriptive cross-sectional study using a mixed-method was carried out with 27 students using a validated satisfaction questionnaire (quantitative data) and conducting scripted interviews (qualitative data) analyzed through the interpretive paradigm. All nursing students expressed a high overall satisfaction with simulated nursing video consultations and positive perceptions about this training program. From the students’ perceptions, three first-level categories and their related second-level and specific categories emerged: belief and myths, skills to improve, and learning improvements. A training program in gender violence victim’s attention through simulated nursing video consultations, in the middle of a pandemic, was a satisfactory experience for nursing students and beneficial for them, as they gained new knowledge and socioemotional skills. This training program mainly improved the acquisition of communication and emotional management skills for an adequate gender violence victim’s attention.

## 1. Introduction

The World Health Organization considers violence against women, or gender-based violence (GBV), as one of the main causes of disability and death for women, and it is also recognized as a public health problem [1,2,3,4]. Intimate partner violence includes stalking, sexual and physical violence, and psychological aggression by intimate male partners or ex-partners [5,6].

The emergence of a novel coronavirus (SARS-CoV-2) in December 2019, which later turned into a worldwide pandemic, also aggravated GBV. To contain the spread of infection, several governments were forced to institute home quarantine and lockdown measures [7]. Consequently, the COVID-19 pandemic, and the social confinement and isolation measures implemented to slow its spread, exacerbated the problem of domestic violence, which increased during this period [8,9].

Whilst data are scarce, reports from across the world, including countries such as China, the United Kingdom, and the United States of America, as well as others, suggested a significant increase in domestic violence cases related to the COVID-19 pandemic [10]. Reports from other countries also indicated a reduction in survivors seeking services due to a combination of lockdown measures and not wanting to visit health services for fear of infection [11].

It should be noted that health professionals, especially nurses, play a fundamental role in GBV, specifically in the early identification, quality of victim care, and prevention of this global public health problem [12]. Specifically, they are often the first contact for these victims, although GBV detection rates are low [13]. Consequently, it is essential to train nurses in GBV to adequately help these victims [14]. In this sense, university students are future health professionals who will be able to care for individuals with this problem from different areas (prevention, detection, and treatment). Therefore, they must be prepared to respond to this type of violence [13,15,16]. It is thus essential to address the education and awareness of these students about this public health problem as a preventive strategy, as health sciences students often do not feel prepared enough to tackle this problem [13,15,16]. Therefore, specific training programs in the area of violence should be promoted, as they produce more proactive attitudes and a higher level of awareness of the problem, especially in women, thus identifying a significant gender effect [17].

However, new training needs in a new context have appeared due to the COVID-19 pandemic. This unprecedented event has forced all the universities around the world to adapt their training programs by adopting new teaching methodologies and different training activities based on the use of information and communication technologies [18].

Within the new teaching methodologies, clinical simulation has been strongly implemented at European universities since the reform of the European Higher Education Area [19], and scientific evidence has been presented about its efficacy for the acquisition of competencies of both clinical and nonclinical skills [20,21]. However, adapting clinical simulation to a virtual context, where the students and the facilitator connect through the Internet, implies an adaptation exercise and a challenge for a new way of developing training processes of virtual simulation, called simulated nursing video consultations [22,23].

The simulated nursing video consultations follows the idea of adapting high-fidelity simulation training to new contexts and with new tools, but it is solely focused on the students [22,23], not on real patients. Using this methodology stimulates reflection and allows for a more comprehensive approach to discover what perceptions the students have about the actions, beliefs, and health practices related to GBV. Specifically, a previous study suggested that this methodology allows nursing students to improve their awareness about the GBV phenomenon, and to acquire a realistic view about their role in GBV care [24]. Therefore, simulated nursing video consultations could help nursing students to improve and promote their acquisition of nontechnical skills (such as active listening, communication skills, empathy, and generation of trust), which are required to adequately care for patients [22,23,24].

It should be indicated that the cultural influence of popular beliefs and myths largely determines the attitudes of nursing students towards GBV. This can increase the risk for the victim and promote tolerance with the aggressors. Thus, specific and transversal con-tents on violence against women need to be introduced in the curricula of nursing degrees, as the training of health professionals is the main strategy for the early detection of violence against women, attendance, and referral, according to the corresponding protocols available to respond to this type of violence [25].

Nevertheless, the use of active methodologies and cooperative learning in GBV has been appropriate for building professional discourses and roles, and for becoming aware and visualizing the possibilities of professional practice. However, students continue feeling unsafe until they can deal with real people, and sometimes, this is not possible in the clinical setting. In light of this, a future line of work was proposed, related to experiential learning opportunities using nonreal patients in simulated scenarios [26].

Therefore, the main aim of the present study was to attain greater knowledge and training of nursing students to deal with GBV cases, and, above all, to achieve its prevention. More specifically, our aims were to describe their satisfaction with this training program, analyze their beliefs about gender violence, to understand the skills that need to be improved, and the posterior effects of a training program in GBV attention through simulated nursing video consultations in Mexican undergraduate nursing students.

## 2. Materials and Methods

### 2.1. Study Design

We conducted a descriptive cross-sectional study using a mixed-method (both quantitative and qualitative data were assessed) to analyze undergraduate nursing students’ satisfaction and perceptions on a training program in GBV attention using simulated nursing video consultations. The qualitative descriptive study was based on the interpretive paradigm [27,28].

### 2.2. Setting and Sample

The study participants were undergraduate students enrolled in a nursing degree at a Mexican public university. All the students were contacted during their participation in simulated video consultations, and they were subsequently interviewed by phone. Convenience criteria were used to choose the group but were not applied for individuals. After that, we continued including students until saturation. The final number of individuals included in the sample was not defined at the beginning of the study, but it was defined during the study process. The final number of informants was 27 Mexican nursing students. The study was conducted between 4 May and 21 May 2020.

### 2.3. High-Fidelity Simulation Procedure

Simulated nursing video consultations based on a potential case of GBV during the COVID-19 confinement were designed using a high-fidelity clinical simulation method-ology. The Blackboard Collaborate Launcher™ was the virtual platform for online video conferences used to carry out this innovative methodology. This virtual platform was provided by each university. The role of a potential GBV victim was played by a standardized patient, who was a woman. To ensure a high level of fidelity experience and a standardized process, this woman was selected and trained to adequately play her role [29]. All simulated nursing video consultations were performed according to the International Nursing Association for Clinical Simulation and Learning (INACSL) Standards of Best Practice: SimulationSM [30,31].

Operational work teams were created (2–3 students), who performed a total of 25 simulated nursing video consultations using a video conference platform and ensuring that each nursing student participated in a video consultation.

- Prebriefing: A prebriefing session was conducted 2 weeks before the performance of the simulated nursing video consultation, establishing a psychologically safe context [32,33] and consolidating the learning process [33]. In this phase, all the students were provided with a short information form about the clinical case (medical history details and health conditions, such as frequent medical consultations, tiredness, feelings of guilt and low self-esteem, headaches, inadequate nutrition, stress due to confinement, and lack of sleep), for them to conduct research on and to collect the existing evidence about the case in advance. All the students were informed that GBV was the main theme of the clinical case proposed. It should be noted that they had been previously trained in the care of GBV victims before the simulated scenario and during the academic year.
- Briefing: To contextualize each simulated nursing video consultation, a brief information session was given to all the students before it took place.
- Simulated scenario: The clinical case was based on a potential GBV victim confined at home during the COVID-19 pandemic, and this situation was simulated to be as realistic as possible. During the simulated scenario, it was necessary to provide the victim with emotional support and to manage her anxiety, as she suffered from an anxiety disorder. All of these nursing interventions are relevant for the early detection and/or adequate management of these victims [34]. The students who performed the simulated nursing video consultations and the standardized patient were at their homes and had an operating microphone and camera installed in their computers.
- Debriefing: After all the simulated nursing video consultations were completed, they were discussed [31]. The debriefing method employed in this phase was the debriefing with a good judgment approach [35]. Furthermore, this phase was structured according to the Gather, Analyze, and Summarize (GAS) debriefing tool [36]. During this debriefing, all the students deeply reflected on their performance during the simulated nursing video consultation, analyzing their positive actions, mistakes, and the actions they need improve in the future.

### 2.4. Data Collection

The Satisfaction Scale Questionnaire with High-Fidelity Clinical Simulation [37] was administrated to collect the quantitative data, determining the satisfaction with the simulated consultations perceived by the nursing students. This questionnaire consists of 33 items and has a five-point response scale (1 = strongly disagree; 5 = totally agree). Its creators obtained a satisfactory internal consistency (Cronbach’s value = 0.920) [37]. On the other hand, the qualitative data, which were collected from an in-depth discussion of relevant topics, was facilitated using a guide consisting of three open-ended questions, which allowed conducting scripted interviews for data collection. These questions were as follows: What myths or beliefs about GBV victim attention have you detected during your performance? What skills do you consider you should improve to provide adequate care for these victims? What have you learnt after the GBV-based simulation session? These questions were obtained both from the categories of the theoretical foundation of debriefing in simulation, and from the theme of gender-based violence [31,35]. These interviews were conducted via the telephone to all the students who agreed to participate voluntarily in the research study 2 days after the last simulated nursing video consultation. All the students were interviewed for 15 min on average by a research team member. The interviewer did not participate in any of the simulated nursing video consultations, thereby avoiding potential bias. It should be noted that this interviewer was a female researcher and nurse lecturer with extensive experience in clinical simulation and qualitative research. After obtaining all the participant’s verbal consents, all the interviews were recorded.

### 2.5. Data Analysis

Firstly, quantitative data were analyzed using IBM SPSS Statistics version 24.0 software (IBM Corp., Armonk, NY, USA), determining the descriptive statistics of both sociodemographic data and items of the questionnaire administrated. Secondly, all students were interviewed, collecting the qualitative data. Two researchers independently analyzed these data. A content analysis of the transcribed interviews was carried out, assisted by the MAXQDA 18 software (VERBI Software, Berlin, Germany). The researchers carried out the data coding, identifying categories and subcategories that emerged from the analysis process, deleting data that provided redundant information, and reviewing data again to find new emergent subcategories [28]. The categories identified at the beginning corresponded with the three open-ended questions asked. Subsequently, new subcategories emerged to describe the students’ perceptions. Finally, the emergent subcategories were saturated during the content analysis process.

### 2.6. Ethical Considerations

We conducted the study accomplishing the ethical principles of the international Declaration of Helsinki [38] for medical research and the data protection and privacy policy currently existing in Spain [39]. Furthermore, the Research and Ethics Board of the Department of Nursing, Physiotherapy, and Medicine at the University of Almeria approved the study with the registration no. EFM 75/2020. We informed all nursing students about the research conducted. The students who agreed to take part voluntarily in the study signed their informed consent. Lastly, we numerically labeled all the participants in chronological order by the day they were interviewed and anonymized them by the letter “S” corresponding to “student”.

## 3. Results

A total of 27 undergraduate nursing students aged between 21 and 28 years old (mean = 22.41; SD = 1.600) participated in our study. Most of the participants were women (*n* = 22; 81.5%). Table 1 shows the descriptive data and frequencies obtained in the analysis of each item contained in the satisfaction questionnaire utilized.

We grouped the five response options of the satisfaction questionnaire into three scales in order to facilitate their analysis since the results obtained were similar (“strongly disagree”/“in disagreement”, “indifferent”, and “in agreement”/“completely agree”). It should be noted that most students obtained scores higher than 90%, scoring mainly in the “in agreement”/“completely agree” scale. In this sense, we obtained the highest scores (100%) for items 6, 8, 11, 17, 18, 19, 23, 27, 28, 29, 30, 31, and the overall satisfaction with the simulation practice (item 33). On the contrary, we obtained the lowest scores for items 13, 16, 22, and 25 (Table 1).

Regarding the qualitative data collected, the results were obtained from the content analysis of the three open-ended questions. Based on the first-level categories of analysis identified (beliefs and myths, skills to improve, and learning needs), various second- and third-level categories were inductively identified (Table 2).

### 3.1. Beliefs and Myths

A worldview of the cultural group regarding GBV was identified, as well as mental representations of this phenomenon.

− There is an important influence of value judgments and prejudices (which interfere with respect and equality, understood as values and fundamental rights of the person).

S28: *“There is a lot of stigma with this issue.”*
S26: *“The question is that women prefer to be assisted by another woman.”*

− Difficulties for therapeutic intervention are commonly perceived. As future health professionals, it is perceived as difficult due to the tendency to overestimate the physical part of the problem (much more than hitting), compared to the other “invisible” dimensions of the person that can be affected by violence.
− Fear and sense of danger if the victim is helped (the victim’s fear radiates and spreads to the people who must activate the help mechanisms). This is an important problem in clinical practice, the perception of threat from the healthcare professional.
− It should be noted that men tended to deny the importance of the GBV phenomenon, showing an important tendency to normalize the gender violence problem. We can observe it direct and indirectly in the next testimonies:

S9: *“Here in Mexico, violence is normal for some people.”*
S25: *“There is not always a justification to fight against gender violence. It is not justified for everyone.”*

In this sense, we found controversial opinions because many students perceived that violence was just one more health problem. Consequently, a great social stigma, a cultural (symbolic) meaning, and a social meaning (associated with the establishment of hegemony/subalternity power relations and which operates structurally at the macro- and microsocial level) were found. However, there was a high degree of social tolerance of gender violence within the institution of marriage and the perception of a lower degree of social help for women who are victims of gender violence, as it was associated with an etiology with an important emotional factor.

S16: *“It only applies from men to women; you have to tolerate it because they are married.”*
S14: *“It is often believed that violence against women does not really exist because the female sex is weak and therefore they are deprived of social benefits or intervention activities because it is a more “sentimental” problem, and details or opinions are hidden in the procedures.”*

In addition, they considered that gender violence only affected less-developed, emerging, or developing countries, and, consequently, perceived a selective appearance of the problem. We can observe that in the next testimony:

S22: *“Gender inequality is not a problem in developed countries.”*

It should be noted that the influence of gender on this problem was also projected on the other side, as shown by health professionals who considered that a battered woman needed a female nursing professional for a complete and real understanding of the problem.

S20: *“Women do not want to be cared for by men and only women suffer gender violence.”*

Finally, the personal meeting in the consultation has always had an important added value regarding telematic consultations, and it was thought that new technologies did not allow addressing and treating gender violence, although it is indeed possible.

### 3.2. Skills to Improve

All the individuals of the sample identified needs for improvement in terms of the following:

− Information and knowledge management on the subject.
− Effective intervention tools (moving forward in the management of the consultation).
− Gaps were identified in the areas of communication, active listening, empathy, and the need for training in carrying out a complete and adequate assessment.
− Working trust also stood out as a main and common point.
− The effective management of value judgments needed improvement, as most participants were aware of the influence that beliefs have on the shaping of judgments and their potential negative or distorting effect.
− Tools to manage emotions: their own, and those of the patients.
− Learning to apply strategies that help and accompany the patient to find that point of internal ambivalence, and to be able to identify for herself that there is something in her life that does not work well and deviates from normality, as the beginning of a therapeutic plan with successful expectations.

In the next two paragraphs, we can find these ideas in some informants’ testimonies; the informants prioritize knowledge, information, and communication, as follows:

S3: *“Empathy and theoretical knowledge about violence.”*
S7: *“More information and how to do the intervention as a nurse.”*
S18: *“what to do if such a situation arises, inform me of the key points to be addressed.”*
S24: *“Know how to give the patient the confidence to share her problem and convince her to speak up.”*
S25: *“I need to improve communication. To have a broad vision of the situation and a better understanding of this issue.”*

In this sense, emphasis was also placed on training at the operational level of the nursing professional, but for preventive purposes. In turn, controversy was manifested in the form of some individuals denying that improvement was needed in this area.

In Mexico, learning about protection mechanisms against personal involvement was demanded; we can observe the importance perceived in testimonies as the following:

S10: *“Learn not to mix my emotions with those of the patient.”*
S18: *“How to establish communication and where the set limit is.”*

At the intervention level, nursing professionals are required to have the operational capacity to refer the patients, as explicitly claimed by several informants:

S11: *“To be able to refer directly the person who is a victim of violence to a protection center.”*
S14: *“To learn more about the subject, how to approach it when presenting a case in which I am involved and ask for help, as well as knowing and having registered contacts of places of support for people who suffer this type of violence. But first of all, how to lose the fear of talking about these issues with those who suffer from them.”*

In addition, improvement needs related to recording of data and follow-up systems were also identified. The continuity in information systems is an important gap found everywhere that represents an important limit for an early intervention. This is a structural deficiency that affects the process and the results.

### 3.3. Learning Improvements

The reflection and critical thinking experienced after remote simulation training allowed students to identify improvements regarding the difficulties found in the therapeutic approach to this problem.

Some general areas of improvement after the experience were also identified as areas to be improved: communication, promoting confidence promotion, prejudice management, and emotions management. 

In addition, nonclinical skills, such as support tools, were also indicated as an important area of improvement in simulated video consultations.

S23: *“That emotional skills and active listening through adequate communication is essential to intervene with patients who suffer this type of violence. And that adequate care must be maintained at all times.”*

The informants underlined an increasing knowledge about the problem (in general), internalizing the advantages of professional training on this topic (therapeutic approach). An example of this improvement area is the following testimony:

S2: *“Violence is not always easy to identify. It is a very important issue that must be addressed, and it can show signs and symptoms that mask the situation. So, it is very important to know them.”*

They also highlighted the preventive character of healthcare professionals when working with gender violence, as well as the skill developed for prioritizing the needs of patients. In addition, they highlighted the progress in identifying gender violence, having learned different manifestations and perceptions according to the culture, the environment, ages, and personal backgrounds. Several testimonies show this idea:

S25: *“There are differences in the perception of gender violence that may be due to culture, geo-graphic location and personal experiences. It is an issue that should be better developed in populations of all ages.”*

In addition, there was a process of awareness regarding the high prevalence of the problem, which facilitated its understanding. Finally, they underlined processes of normalization of the situation, from which it was assumed that gender violence is not a problem.

S11: *“I have learned that you always have to start by maintaining the patient’s confidence in herself, to understand the value that she has and to convince her to get help for her personal situation.”*
S8: *“This is very usual; it is part of the way we relate to each other.”*

Those testimonies of denial of learning corresponded to the behaviors of normalization of violence and to a set of beliefs justified with dynamics and violent ways of living. Denial of learning was manifested mainly by male informants. When someone normalizes the existence of a problem, they nullify any need to learn how to deal with that situation, and they recognize even less that they need to improve.

## 4. Discussion

The current pandemic situation has become an important source of anxiety, anguish, and added stress that is affecting everyone’s health [40]. In addition, these feelings are combined with the specific circumstances of confinement at home where GBV is a reality, and which has increased during this critical world crisis [7,8,9,10,11]. Health professionals, doctors, and nurses play a fundamental role in the prevention and healthcare intervention in GBV [41]. Specifically, nurses are essential professionals in the early identification, quality of victim care, and prevention [12]. In this sense, they are often the first contact to these victims [13]. This is the reason for this study, which was conducted to analyze the satisfaction with a training program, based on simulated video consultations and attitudes of nursing students, to better understand their beliefs and myths related to gender-based violence, as well to analyze their perception about the skills needed to be improved and those aspects that had already improved after the training program, based on simulated video consultations.

Our quantitative results indicate a high satisfaction with this training program, supported by the overall satisfaction with the simulated sessions expressed by nursing students (100%). Other advantages expressed were related to the opportunity to learn to avoid mistakes, learn from errors, reinforce critical thinking and decision-making, assess patients’ conditions, prioritize care, and improve communication with the patient. These results are consistent with other studies that achieved positive learning outcomes and a high level of satisfaction from learners using clinical simulation methodology [36,42,43,44]. Specifically, our nursing students realize the relevant role of the debriefing phase in their learning, with this result being congruent with other studies [45,46]. In addition, the nursing students highlighted the practical usefulness of the simulated sessions conducted (96.3%). In this sense, the increased use of telemedicine, specifically the emergent use of video consultations [47], has been prompted by the COVID-19 pandemic, challenging all healthcare systems worldwide. Consequently, it is necessary to train and educate all healthcare professionals in this healthcare modality to provide high-quality care, properly managing a video consultation [48].

However, learners often expressed high levels of anxiety during clinical simulation sessions in previous studies [49,50]. By contrast, our students expressed calmness during the simulated scenarios (74%). In this sense, current recommendations and standards defined by the literature were followed to create a safe environment during simulated scenarios in our study [32,33]. In addition, our students perceived technical skills and communication with family development as disadvantages of this training program. In contrast to previous studies in clinical simulation [20,21], the results obtained indicated that this simulation methodology was not adequate to develop clinical skills. However, this issue has also been indicated in real clinical situations, as healthcare professionals complain about the impossibility of performing clinical procedures, techniques, or physical examinations through video consultations [51,52]. Furthermore, our students expressed that the simulations did not improve communication with the family. This may be because not all simulated nursing video consultations required this type of communication.

Regarding our qualitative results, and according to Coll-Vinent et al. [53], the fact that a health professional does not recognize gender-based violence as a health problem implies a great difficulty for detecting and treating the problem, which is mainly due to the lack of training and knowledge in this respect [54,55]. The common reference framework among the students coincided in diverse aspects such as denial, by the men, of previous ideas about gender-based violence; the influence of value judgements and prejudices; the difficulty for intervention, the trend in overestimating the physical part of the problem; awareness or identification of the problem by the patient; and fear and sense of danger if the victim is helped.

As for the differences between men and women, studies have shown differences in male chauvinistic beliefs sustained about gender-based violence [55], and although progress has been made with the youth for overcoming machismo, it is still very much present [56], with this overcoming being a step forward for women, but not always valued the same by the men, who tend to perceive it more as a loss rather than a gain [57]. In our study, the gender factor had an influence on the perception of violence. Therefore, gender was manifested as an aspect that prevailed even when all the individuals belonged to the same group: health professionals. As for gender-based violence, it was evidenced that being a man prevailed over being a healthcare worker when believing in myths and having beliefs with respect to how to address the problem.

Among the beliefs and myths that appeared between the youth with respect to gender-based violence, we found a tendency to normalize the problem (although the youth are beginning to consider it a health problem); they indicated a great social stigma and a high degree of social tolerance within the marriage (male domination or female inferiority is something that is imprinted in the sociocultural environment), they believed that it greatly affected less-developed countries, and also considered that a female nurse would better understand a patient who suffered gender-based violence, and also did not consider the new technologies as a resource that could be used to address the problem.

It should be taken into account that in Mexico, women tend to be frequently devalued, forming part of subordinate groups and not always receiving the adequate attention from the law. Therefore, this phenomenon constitutes a male chauvinistic reaction that influences the minimization of the importance of the different forms of gender violence [58]. Latin America and the Caribbean are the regions of the world with the most cases of violence against women [59], and with the lockdown measures due to COVID-19, the help lines have observed an increase [60].

Beliefs that are more tolerant with gender-based violence are important factors of risk for the appearance of this type of violence. The more traditional beliefs about the role of women attributes them with a greater responsibility in the conflicts between partners than when there is a less traditional point of view [17].

The students identified subjects which needed to be improved: knowledge (and gaining trust), intervention tools, communication and emotional skills, aid-based relationship, and the better management of value judgement. More specifically, our informants considered that they had to improve the management of emotions as opposed to personal involvement, opportune knowledge for the referral, and the system of records and monitoring of the cases. 

Among the improvements in learning recorded, as produced by the training with simulated video consultations, some were also present in the “need to be improved” list that was previously mentioned. These were communication and emotional management, the management of value judgements, and the increase in confidence and competencies. Among the men, we found some testimonies of denial of learning which answered to the normalization of the problem, with the risk this implies for conducting an adequate intervention in gender violence. In addition, as an improvement, the students highlighted the increase in knowledge and training, together with an increase in awareness, with what this implies for the prevention and the toppling of prejudices. Let us remember that the corresponding protocols available to respond to gender violence indicate the need for training on GBV to adequately help these victims [14,24] and after the results obtained, we consider that this type of training (clinical simulation) can be a great strategy to improve early detection of violence against women, care, and referral.

The scientific evidence indicates the importance of offering adequate training for creating favorable attitudes towards an adequate intervention against gender-based violence [23,61]. The attitudes improved when there was specific training on this respect [61]. In addition, the simulated video call allows developing a more critical attitude about the problem, resolving any prejudice that could be observed towards the future patient in advance.

Some studies have indicated that university training about gender issues does not seem to have an influence on male chauvinistic beliefs [61]; a wager is made then, aside from transmitting a nonmale chauvinistic model of relationships, for specific contents that could at least improve the attitude towards this great problem of public health, because, as indicated by Macías Seda et al., when training is available, differences appear between the youth who have received training (who are more sensitized) as compared to those who have not received it, although this difference does not appear in men [61].

According to a study by Arredondo et al., receiving information is positively related with greater knowledge about a subject [62]. This training could allow health professionals to conduct an earlier detection of the abuse, with the reduction of the consequences at the level of health and even healthcare spending [63].

Also, this training should include strategies destined to provide an answer to the violence before and after the pandemic. These strategies should be based on what was learned in prior emergency situations [64]. 

Among the limitations of the study, and given that the study utilized new technologies, and virtual healthcare, we increased our awareness of the possible dangers of technology due to the dehumanization of individuals, and the prioritization of technology over the person. Thus, there is a need for the program implemented with this technology to be continuously tested and renewed. In this sense, more research on humanization of healthcare using video consultations is needed. Another limitation was not being able to extrapolate the results to other populations, as well as the reactivity of the qualitative studies themselves. Regarding possible participation bias, the availability of video calling in virtual learning environments, as well as the context of confinement, could increase the risk for this bias. However, theoretical triangulation and triangulation of researchers were carried out during our research, avoiding these possible biases. Finally, the results obtained in our study should be treated cautiously since we address the perceptions of a specific sample. Thus, more research on this topic is recommended to further compare the perceptions on GVB.

## 5. Conclusions

A training program in gender violence victim’s attention through simulated nursing video consultations, in the middle of a pandemic, was a satisfactory experience for nursing students who participated in these simulation sessions. This training program was beneficial for nursing students, as they gained new knowledge and socioemotional skills. The students highlighted the awareness of the problem. Specially for the men, a framework appeared, related to the denial of the previous ideas about gender-based violence, which urges us to intervene and provide more education about this serious health problem. Gender prevails over the student’s membership to the health-related professions, and on the perception and mental representations of GBV.

The nursing students showed a tendency to normalize the problem, but among the aspects that were improved, as previously mentioned, we found a greater awareness. As for the areas that needed to be improved, they indicated the knowledge needed about the subject matter, and the need for tools for the adequate creation of an aid-based relationship. The students demanded protection against their emotional involvement and a greater recording of the cases.

All of this information compels us to pay more attention to the training offered, as related to the content and the manner of providing it. The contexts of simulation and new technologies allow us to assess content and actions related to equality, violence, empathy, emotional intelligence, respect, and proactivity, as observed in our study.

It is necessary to insist on this manner of educating and to observe the differences between men and women related to the subject of violence. We did not find homogeneity between the sexes, and thus, we could develop and invest more in better training efforts that are closer to the reality we are currently living in during the pandemic and its related increase in violence in the family and home environments. In addition, there was little scientific evidence regarding training actions, and even less regarding the use of new technologies within them. Therefore, we recommend continuing to investigate in this research line in the future.

## Figures and Tables

**Table 1:** Descriptive data and frequencies obtained in our sample (*n* = 27) in each item included in the satisfaction questionnaire.

Item	Mean (SD)	Strongly Disagree/In Disagreement	Indifferent	In Agreement/Totally Agree
1. Facilities and equipment were real.	4.04 (0.980)	11.1%	11.1%	77.8%
2. Objectives were clear cases.	4.70 (0.669)	3.7%	0%	96.3%
3. Cases recreated real situations.	4.81 (0.622)	3.7%	0%	96.3%
4. Timing for each simulation case was adequate.	4.26 (0.712)	3.7%	3.7%	92.6%
5. The degree of cases difficulty was appropriate to my knowledge.	4.59 (0.694)	3.7%	0%	96.3%
6. I felt comfortable and respected during the sessions.	4.89 (0.320)	0%	0%	100%
7. Clinical simulation is useful to assess a patient’s clinical simulation.	4.70 (0.669)	3.7%	0%	96.3%
8. Simulation practices help you learn to avoid mistakes.	4.78 (0.430)	0%	0%	100%
9. Simulation has helped me to set priorities for action.	4.52 (0.753)	0%	14.8%	85.2%
10. Simulation has improved my ability to provide care to my patients.	4.11 (1.103)	7.4%	11.1%	81.5%
11. Simulation has made me think about my next clinical practice.	4.89 (0.320)	0%	0%	100%
12. Simulation improves communication and teamwork.	4.60 (0.797)	3.7%	7.4%	88.9%
13. Simulation has made me more aware/worried about clinical practice.	3.22 (1.038)	44.4%	29.6%	26%
14. Simulation is beneficial to relate theory to practice.	4.63 (0.565)	0%	3.7%	96.3%
15. Simulation allows us to plan the patient care effectively.	4.48 (0.753)	3.7%	3.7%	92.6%
16. I have improved my technical skills.	3.89 (0.974)	11.1%	7.4%	81.5%
17. I have reinforced my critical thinking and decision-making.	4.56 (0.506)	0%	0%	100%
18. Simulation helped me assess patient’s condition.	4.30 (0.669)	3.7%	0%	100%
19. This experience has helped me prioritize care.	4.48 (0.509)	0%	0%	100%
20. Simulation promotes self-confidence.	4.56 (0.577)	0%	3.7%	96.3%
21. I have improved communication with the team.	4.15 (0.662)	0%	14.8%	85.2%
22. I have improved communication with the family.	3.71 (0.912)	3.7%	37%	59.3%
23. I have improved communication with the patient.	4.52 (0.509)	0%	0%	100%
24. This type of practice has increased my assertiveness.	4.37 (0.629)	0%	7.4%	92.6%
25. I became nervous during some of the cases.	2.22 (1.013)	63%	29.6%	7.4%
26. Interaction with simulation has improved my clinical competence.	4.22 (0.801)	3.7%	11.1%	85.2%
27. The teacher gave constructive feedback after each session.	5.00 (0.000)	0%	0%	100%
28. Debriefing has helped me reflect on the cases.	5.00 (0.000)	0%	0%	100%
29. Debriefing at the end of the session has helped me correct mistakes.	4.93 (0.267)	0%	0%	100%
30. I knew the cases’ theoretical side.	4.74 (0.447)	0%	0%	100%
31. I have learned from the mistakes I made during the simulation.	4.70 (0.465)	0%	0%	100%
32. Practical utility.	4.81 (0.483)	0%	3.7%	96.3%
33. Overall satisfaction with the sessions.	4.93 (0.267)	0%	0%	100%

**Table 2:** Comprehensive list of categories identified after content analysis.

1st Level Categories	2nd Level Categories	Specific Categories—3rd Level
1. Beliefs and myths	Prejudice	Social stigma
	Denial	Cultural meaning (symbolism)
	Intervention difficulties	Social sense
	Awareness	Normalization and tolerance
	Fear	Less social production of help related to emotional etiology factors
		Selective appearance
		New technologies limitations
2. Skills to improve	Knowledge and information management	Operational intervention capacity
	Intervention tools	Personal protection
	Accompaniments	Recording and follow-up of cases
	Communication ^1^	
3. Learning improvements	Confidence promotion ^1^	Awareness
	Prejudice management ^1^	Normalization
	Emotions management ^1^	Early identification
	Denial of learning	General knowledge of the topic
	Supportive tools	Personal prevention
	Violence normalization	

^1^ The general second-level categories highlighted in gray are skills to improve as learning improvements.

## Data Availability

The data presented in this study are available on request from the corresponding author.
